# Imagerie des tumeurs brunes maxillo-mandibulaires multiples: à propos d’un cas

**DOI:** 10.11604/pamj.2021.38.4.27141

**Published:** 2021-01-05

**Authors:** Asmaa Adnane, Aicha Merzem, Meryem Harmak, Hasnaa Belgadir, Omar Amriss, Naima Moussali, Naima Elbenna

**Affiliations:** 1 Département de Radiologie, Faculté de Médecine et de Pharmacie, Université Hassan II Casablanca, Casablanca, Maroc

**Keywords:** Tumeur brune, face, insuffisance rénale chronique, hyperparathyroïdie, *case report*, Brown tumor, face, chronic renal failure, hyperthyroidism, case report

## Abstract

Les tumeurs brunes sont des lésions ostéolytiques rares, survenant chez 1,5 à 1,7% des patients en insuffisance rénale chronique terminale. Elles sont le produit d´un remodelage osseux sous l´effet de la parathormone. Nous rapportons l´observation d´une jeune femme suivie pour insuffisance rénale chronique terminale sous hémodialyse et qui présentait des tuméfactions maxillo-mandibulaires indolore apparues 7 mois avant sa consultation. L´examen clinique a retrouvé une déformation faciale avec à la palpation deux masses maxillaires et mandibulaires. Le bilan biologique a révélé une hypocalcémie, une hyperphosphatémie avec une hyperparathyroïdie. Le scanner de la face a révélé de multiples masses ostéolytiques au niveau de l´os maxillaire et de la mandibule avec importante raréfaction de la trame osseuse et des signes de résorption osseuse diffuse. Le diagnostic de tumeurs brunes multiples de la face a été retenu sur un faisceau d´arguments cliniques, biologiques et radiologiques. Les tumeurs brunes sont une entité rare dont l´atteinte maxillo-mandibulaire est fréquente chez l´insuffisant rénale chronique. Elle doit être connue du praticien d´où l´intérêt de cette observation.

## Abstract (en)

Brown tumors are rare osteolytic lesions occurring in 1.5-1.7% of patients with chronic end-stage renal failure. They are caused by PTH-induced bone remodeling. We here report the case of a young woman on hemodialysis for chronic end-stage renal failure with painless maxillo-mandibular swellings occurred 7 months before. Clinical examination showed facial deformity with two maxillary and mandibular masses to palpation. Laboratory tests revealed hypocalcemia, hyperphosphatemia with hyperparathyroidism. CT scan of the face revealed multiple osteolytic masses in the maxillary and mandibular bones, with significant bone rarefaction and signs of diffuse bone resorption. The diagnosis of multiple brown tumors of the face was retained based on clinical, biological and radiological features. Brown tumors are a rare entity usually characterized by maxillomandibular involvement in patients with chronic renal failure. Practitioners should suspect them, hence the interest in this study.

## Introduction

Les tumeurs brunes appelées également ostéite fibreuse de Von Recklinghausen sont des lésions bégnines et non néoplasiques. Elles sont rares et touchent entre 1,5 et 1,7% des patients atteints d´insuffisance rénale chronique terminale. Elles résultent d´une hyperparathyroïdie secondaire à une carence de synthèse de la vitamine D dans le rein responsable d´une lyse avec remodelage osseux. Il s´agit d´une entité rare où l´atteinte maxillo-mandibulaire est fréquente et doit être connue du praticien. Nous rapportons l´observation d´une jeune femme suivie pour insuffisance rénale chronique terminale sous hémodialyse et qui présente des masses multiples de la face.

## Patient et observation

**Figure 1:**
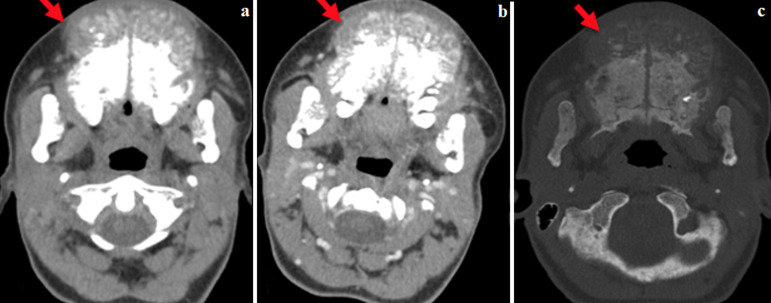
TDM de la face, coupes axiales ; a et b) fenêtre partie molle sans et avec injection de PDC, c) fenêtre osseuse: masse maxillaire ostéolytique, tissulaire, rehaussée après injection de PDC

**Figure 2:**
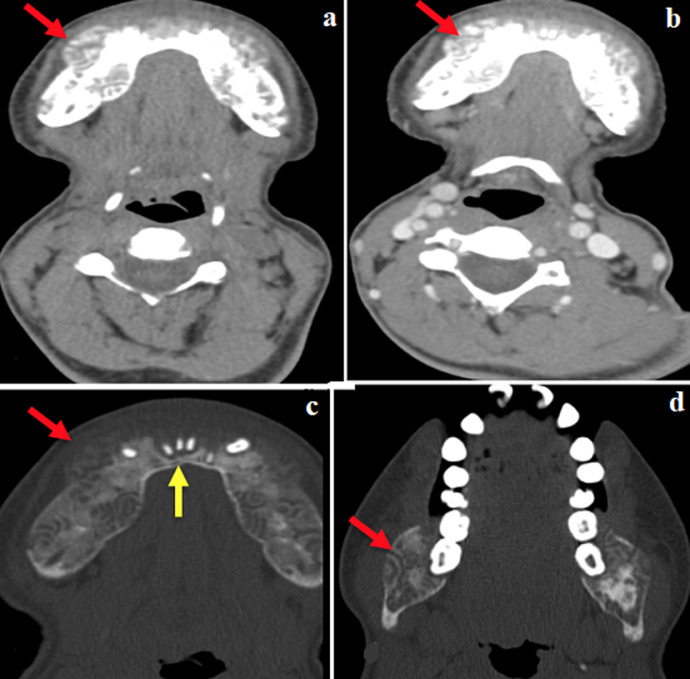
TDM de la face, coupes axiales ; a et b) fenêtre partie molle sans et avec injection de PDC, c et d) fenêtre osseuse: masses ostéolytiques de la symphyse mandibulaire et des ramus mandibulaires, de densité tissulaire, rehaussées après injection de PDC avec lyse de la lamina dura et de l´os alvéolaire en regard des incisives inférieures

Il s´agit d´une jeune femme de 28 ans, ayant comme antécédent une insuffisance rénale chronique terminale sur polykystose rénale héréditaire bilatérale, sous hémodialyse à raison de 2 séances par semaine. La patiente se plaignait de tuméfactions maxillaire et mandibulaire apparues 7 mois avant sa consultation. Elle ne rapportait pas de douleur mais présentait une gêne à la mastication. L´examen clinique a retrouvé deux masses maxillo-mandibulaires, dures et fixes par rapport au plan profond, avec une mobilité des incisives inférieures. Le bilan phosphocalcique était perturbé avec une hypocalcémie, une hyperphosphatémie et un taux de parathormone élevé. Devant la présence de la tuméfaction maxillo-mandibulaire, un scanner de la face a été réalisé sans et avec injection de produit de contraste. Le scanner a objectivé la présence au niveau de l´os maxillaire, de la symphyse mandibulaire et des ramus mandibulaires de multiples masses ostéolytiques, ovalaires, assez bien limitées, de densité tissulaire, rehaussées après injection de produit de contraste, sans réaction périostée ni envahissement des structures adjacentes (Figure 1, Figure 2).

**Figure 3:**
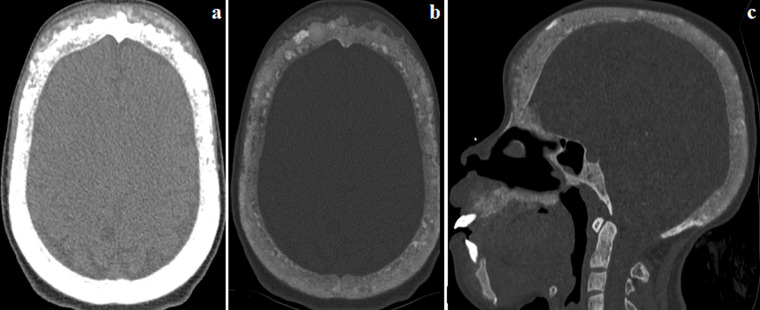
TDM de la face ; a) coupe axiale en fenêtre partie molle, b) coupe axiale en fenêtre osseuse, c) reconstruction coronale en fenêtre osseuse: déminéralisation osseuse diffuse du scalp avec pseudo-élargissement et dédifférenciation table-diploé réalisant l´aspect en « sel et poivre » témoignant de la résorption endostée

**Figure 4:**
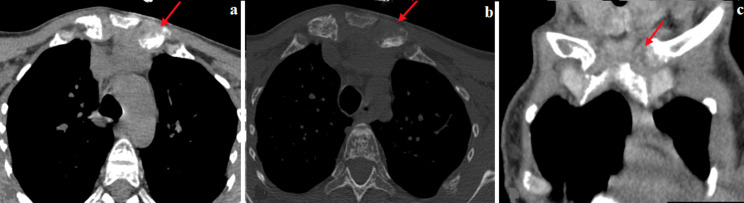
TDM thoracique ; a) coupe axiale fenêtre médiastinale, b) coupe axiale fenêtre osseuse, c) reconstruction coronale fenêtre médiastinale: élargissement de l´interligne articulaire sterno-claviculaire gauche

La masse de la symphyse mandibulaire était responsable d´une lyse de la lamina dura et de l´os alvéolaire des dents en regard réalisant l´aspect de «dents flottantes» (Figure 2). Nous avons noté également la présence d´une importante déminéralisation des os de la face et du scalp siège d´un pseudo élargissement avec un aspect irrégulier de ses contours et une dédifférenciation table-diploé réalisant un aspect «sel et poivre» en rapport avec une résorption endostée (Figure 3). Sur les coupes thoraciques, nous avons objectivé un élargissement de l´interligne sterno-claviculaire gauche en rapport avec une résorption sous chondrale (Figure 4). Nous n´avons pas noté d´hyperplasie des parathyroïdes. Le diagnostic de tumeurs brunes sur ostéodystrophie rénale a été retenu devant le contexte d´insuffisance rénale chronique terminale avec une hyperparathyroïdie secondaire au long cours et l´aspect radiologique. Le traitement était médicamenteux. Vu l´absence d´une hyperplasie des parathyroïdes, la chirurgie n´était pas indiquée.

## Discussion

Les tumeurs brunes sont des lésions rares, survenant chez 1,5 à 1,7% des patients en insuffisance rénale chronique (IRC) terminale [1]. Elles sont bénignes et non néoplasiques résultant d´un remodelage osseux sous l´effet de la PTH où le tissu osseux normal est remplacé par du tissu conjonctif, de l´hémorragie et de l´hémosidérine [2]. L'hyperparathyroïdie secondaire représente un trouble courant chez les patients atteints d'IRC. Elle se développe à la suite d'une hyperphosphatémie, d'une hypocalcémie et d'une altération de la synthèse rénale de la vitamine D avec réduction des taux sériques de calcitriol. L´hyperparathyroïdie tertiaire, plus rare est une conséquence de l´hyperparathyroïdie secondaire de longue durée qui, en raison d'une insuffisance rénale, développent un fonctionnement autonome de la parathyroïde avec hypersécrétion de PTH [3]. Certains auteurs défendent également l'existence d'un type quaternaire, où l'hyperplasie autonome de l'hyperparathyroïdie tertiaire évolue vers la formation d'adénomes parathyroïdiens [4]. Les lésions peuvent être uniques ou multiples et peuvent intéresser tous les os.

Les symptômes dépendent de leur taille et de leur localisation [5]. Cliniquement, elles sont le plus souvent asymptomatiques. Les tumeurs brunes de la face sont responsables de déformation faciale avec gêne et altération de la mastication [6] comme c´est le cas chez notre patiente. En imagerie, les tumeurs brunes apparaissent comme des lésions ostéolytiques bien définies, siège de formations denses et hétérogènes au sein de la nouvelle structure, avec hypertrophie osseuse particulièrement nette à la mandibule lorsqu´elles s´associent à une ostéite fibro-kystique [7].

Les changements dentaires rapportés en association avec l´HPT comprennent des chambres pulpaires anormalement étroites, une résorption de la lamina dura autour des racines des dents et une déminéralisation des os médullaires des mâchoires provoquant un aspect en «verre dépoli» caractéristique [8]. L´ensemble de ces anomalies radiologiques ont été retrouvées chez notre patiente. Le diagnostic de tumeurs brunes multiples de la face était évident chez notre patiente devant le contexte clinique d´insuffisance rénale avec hyperparathyroïdie secondaire et l´aspect radiologique évocateur. Aucun autre diagnostique n´a été soulevé. Les tumeurs brunes présentent histologiquement l´aspect d´une lésion à cellules géantes. En dehors d´un contexte d´hyperparathyroïdie connu, le diagnostic différentiel se pose donc avec les granulomes réparateurs à cellules géantes et les tumeurs à cellules géantes. Le traitement est médical dans la majorité des cas et vise à normaliser le taux de la parathormone. La parathyroïdectomie est indiquée lorsque la maladie est résistante au traitement médical [9]. La normalisation des taux de PTH entraînera souvent une régression des tumeurs brunes ou parfois même une résolution spontanée [10].

## Conclusion

L´hyperparathyroïdie secondaire et tertiaire sont fréquentes chez les patients suivis pour une insuffisance rénale chronique terminale. Il en résulte un processus de remodelage osseux aboutissant à la formation de tumeurs brunes. Cette observation souligne l´intérêt de l´imagerie qui en connaissance du contexte clinique et biologique; permet de faire le diagnostic positif. Ceci permet d´exclure les autres diagnostics différentiels, épargnant aux malades une chirurgie inutile de ces lésions.
